# Anodal tDCS over left parietal cortex expedites recovery from stroke-induced apraxic imitation deficits: a pilot study

**DOI:** 10.1186/s42466-019-0042-0

**Published:** 2019-11-26

**Authors:** Jana M. Ant, Eva Niessen, Elisabeth I. S. Achilles, Jochen Saliger, Hans Karbe, Peter H. Weiss, Gereon R. Fink

**Affiliations:** 1 Department of Neurology, Faculty of Medicine and University Hospital Cologne, University of Cologne, Cologne, Germany; 2 Cognitive Neuroscience, Institute of Neuroscience and Medicine (INM-3), Research Center Jülich, 52425 Jülich, Germany; 3 Neurological Rehabilitation Centre Godeshöhe, Bonn, Germany

**Keywords:** Apraxia, Neuromodulation, Stroke, Neurorehabilitation, Transcranial direct current stimulation (tDCS), Motor cognition

## Abstract

**Background:**

To date, specific therapeutic approaches to expedite recovery from apraxic deficits after left hemisphere (LH) stroke remain sparse. Thus, in this pilot study we evaluated the effect of anodal transcranial direct current stimulation (tDCS) in addition to a standardized motor training on apraxic imitation deficits.

**Methods:**

In a rehabilitation hospital, we assessed apraxic, aphasic, and motor deficits in 30 LH stroke patients before and after a five-day standard programme of motor training combined with either anodal (10 min, 2 mA; *n* = 14) or sham (10 min, 0 mA, *n* = 16) tDCS applied in a double-blind fashion over left posterior parietal cortex (PPC). Where appropriate, data were analyzed with either t-test, Fisher’s exact test, or univariate/ repeated measures ANOVA.

**Results:**

Compared to sham tDCS, five sessions of anodal tDCS expedited recovery from apraxic imitation deficits (*p* < 0.05): Already after 5 days, the anodal tDCS group showed levels of imitation performance that were achieved in the sham tDCS group after 3 months. However, the primary outcome of the study (i.e., anodal tDCS induced improvement of the total apraxia score) failed significance, and there was no significant tDCS effect on apraxia after 3 months. Anodal tDCS improved grip force (of the contra-lesional, i.e., right hand), but had no effect on aphasia.

**Conclusions:**

Data from this pilot study show that repetitive, anodal tDCS over left PPC combined with a standardized motor training expedites recovery from imitation deficits in LH stroke patients with apraxia (relative to sham stimulation). Results suggest that in patients suffering from apraxic imitation deficits a randomized controlled trial (RCT) is warranted that investigates the effects of tDCS applied over PPC in addition to a standardized motor training.

## Background

Apraxia is a disorder of motor cognition leading to deficits in imitation, pantomiming, and tool use that cannot solely be explained by paresis or sensory deficits [36]. It is most commonly observed after left hemisphere (LH) damage. Despite the facts that i) apraxic upper limb deficits occur in up to 50% of the patients with a LH stroke [5, 41], and, importantly, ii) they constitute a poor prognostic factor for neurorehabilitation [22], specific therapeutic options for apraxic deficits to date remain sparse [13].

Depending on the direction of the current, transcranial direct current stimulation (tDCS) can modulate cortical excitability [37] and thereby aggravate or ameliorate neurological deficits [16]. To date, however, clinically relevant evidence for the effectiveness of tDCS for improving performance of activities of daily living (ADL) after stroke is of low to moderate quality [14].

Apraxic impairments of the imitation of gestures and pantomiming are frequently observed after LH stroke [23]. Although the pathophysiology of apraxic pantomime deficits is still a matter of debate [32], the pivotal role of the left PPC for (hand) gesture imitation is undisputed [12, 20]. Consistent with such a critical role, single sessions of anodal tDCS over the left posterior parietal cortex (PPC) facilitated gesture processing in healthy subjects [43] and improved the performance of apraxic patients (*n* = 6) in imitation tasks involving the upper limb [3].

Accordingly, in this pilot study, we assessed whether in combination with a standardized motor training anodal (versus sham) tDCS applied over left PPC expedites recovery from apraxic deficits.

Based on previous work [43], we expected that (only) anodal tDCS over the PPC facilitates recovery from apraxic deficits and in particular apraxic imitation deficits [1], since anodal tDCS leads to increased excitability within the stimulated cortex (here: PPC [37];) and has been shown to temporarily ameliorate deficits in hand gesture imitation [3]. For this reason and due to the reduced resilience of the LH stroke patients, we here focused on anodal (versus sham) tDCS above the PPC. Note that previous studies employing anodal, sham, and cathodal tDCS above different stimulation sites (right/left PPC, right/left primary motor cortex, M1) also found positive effects on gesture processing only for 2 mA anodal tDCS above left PPC [3, 7].

To this end, we included in this study 30 sub-acute LH stroke patients, who at the time of testing were undergoing their regular patient-tailored rehabilitation program including physiotherapy, occupational therapy, speech therapy and neuropsychological therapy in an in-patient setting (rehabilitation hospital).

## Methods

### Participants

**Fig. 1:**
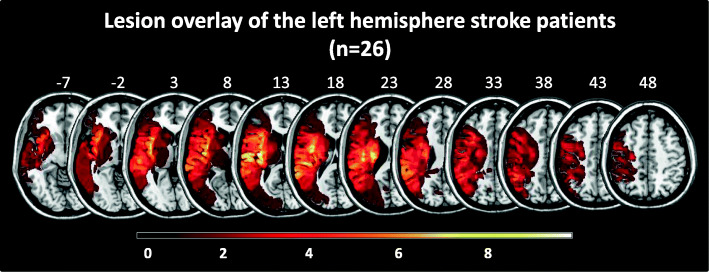
Lesion distribution in the current sample of left hemisphere (LH) stroke patients (*n* = 26). Note that for four patients no scan suitable for lesion mapping was available. Color shades represent the increasing number of overlapping lesions. Slices with the MNI-z-coordinates from − 7 to 48 are shown

We screened 67 patients undergoing neuro-rehabilitation after a first-ever ischemic LH stroke. Of those, 30 right-handed LH stroke patients (59.1 ± 12.8 years old, 76.5 ± 132.2 days post-stroke at baseline assessment (median: 24.3 days), 5 women) met the inclusion criteria (no previous history of neurological or psychiatric diseases, no alcohol or drug abuse, sufficient knowledge of German, right-handedness, no metal implants in the head/neck region, and no cardiac pacemaker). Premorbid handedness was assessed by the Edinburgh Handedness Inventory [35]. Note that all patients had a laterality quotient (LQ) ≥ + 80, i.e., were in or above the 5th decile for right-handedness. We used the Mini-Mental State Examination (MMSE, cut-off: < 24 of 30 points, [8]) and the Hospital Anxiety and Depression Scale (HADS, cut-off for anxiety: < 8 of 21 points, cut-off for depression: < 8 of 21 points, [47]) to exclude clinically relevant cognitive decline, anxiety, or depression. The modified Rankin Scale (mRS) revealed a mild to moderate disability [38]. Table 1 summarizes the demographic, clinical, and neuropsychological data of the 30 LH stroke patients. Figure 1 depicts the lesion distribution of the current sample of left hemisphere (LH) stroke patients. Note that for four patients, no clinical MRI or CT scan suitable for lesion mapping was available (no consent: *n* = 1, only initial CT scans available with no demarcated stroke lesion despite persistent deficits: *n* = 3). Lesion mapping was based on clinical imaging by CT (*n* = 12) or MRI (*n* = 14; see also [45]). Using the MRIcron software (http://people.cas.sc.edu/rorden/mricron/index.html), an investigator (JMA or EN) manually transferred the lesions to a T1-weighted template brain (ch2.nii). The lesion mapping was double-checked by the other investigator (EN or JMA); both investigators had to agree on lesion location and extent.

**Table 1:** Demographic, clinical and neuropsychological data of the LH stroke patient groups undergoing anodal or sham tDCS (applied over left posterior parietal cortex, PPC)

	Anodal group *n* = 14	Sham group *n* = 16	Statistical parameters of the group comparisons
Age (*years*)	58.4 (±13)	59.8 (±13)	t(28) = .305, *p* = .762
Gender (*female/male*)	1/13	4/ 12	*X*^2^(1) = 1.714, *p* = .336
Time post stroke (*median in days*)	25.5	23.0	t(28) = .751, *p* = .459
Days till follow-up	100.9 (±7.0)	99.4 (±10.7)	t(23) = −.396, *p* = .696
MMSE (*max. 30*)	28.0 (±1.5)	28.3 (±0.9)	t(21) = .604, *p* = .552
HADS Anxiety score (*max. 21, cut-off > 7*)	5.2 (±3.5)	4.3 (±3.8)	t(26) = −.641, *p* = .527
HADS Depression score (*max. 21, cut-off > 7*)	5.0 (±3.1)	3.7 (±2.7)	t(26) = − 1.157, *p* = .258
LQ	96.0 (±8.0)	95.3 (±8.5)	t(28) = −.248, *p* = .806
MRC paresis scale (*right hand, max. 5*)	3.8 (±2.1)	4.6 (±0.5)	t(28) = 1.447, *p* = .169
Relative grip force of the contra-lesional right hand (%, in relation to the ipsi-lesional left hand)	65.8 (±10.1)	78.0 (±7.4)	t(28) = .981, *p* = .335
mRS	2.5 (±1.2)	2.0 (±1.0)	t(28) = −1.165, *p* = .254
ACL-K (*max. 40, cut-off < 33*)	25.4 (±11.5)	28.8 (±10.7)	t(28) = .837, *p* = .410
KAS total score (*left hand, max. 80, cut-off < 77*)	70.6 (±8.6)	70.4 (±13.5)	t(28) = −.047, *p* = .963
KAS pantomime score (*max. 40*)	36.1 (±5.0)	35.7 (±4.5)	t(28) = −.263, *p* = .794
KAS imitation score (*max. 40*)	34.4 (±4.8)	34.4 (±9.2)	t(28) = −.019, *p* = .985
De Renzi actual object use test (*left hand, max. 32*)	31.4 (±0.9)	31.0 (±2.0)	t(28) = −.744, *p* = .463

Given are the means and the standard deviations from the mean (SD, in parenthesis; if not stated differently). There were no significant differences between the anodal and the sham patient groups for any variable (all *p* > .1)

*LH* left hemisphere, *tDCS* transcranial direct current stimulation, *PPC* posterior parietal cortex, *MMSE* Mini Mental State Examination, *HADS* Hospital Anxiety and Depression Scale, *LQ* Laterality quotient as assessed by the Edinburgh Handedness Inventory, *MRC paresis scale* Medical Research Council rating scale for assessing paresis, *mRS* modified Rankin scale, *ACL-K* Aphasia Check List-short version, *KAS* Cologne Apraxia Screening

A person who was not involved in the assessment or tDCS stimulation of the LH stroke patients assigned the patients consecutively to either the anodal (n = 14) or sham (*n* = 16) tDCS groups, which were eventually matched for age and time post-stroke as well as the severity of apraxia, basic motor (dys-)function, and aphasia at baseline assessment (see Table 1). Both stimulation groups comprised LH stroke patients with and without apraxia (anodal tDCS: 9 apraxic and 5 non-apraxic patients, sham tDCS: 11 apraxic and 5 non-apraxic patients).

All patients gave written informed consent before participating in the study. The study was carried out following the ethical principles of the World Medical Association (Declaration of Helsinki) and after obtaining approval by the ethics committee of the Medical Faculty in Cologne.

### Study description and design

**Fig. 2:**
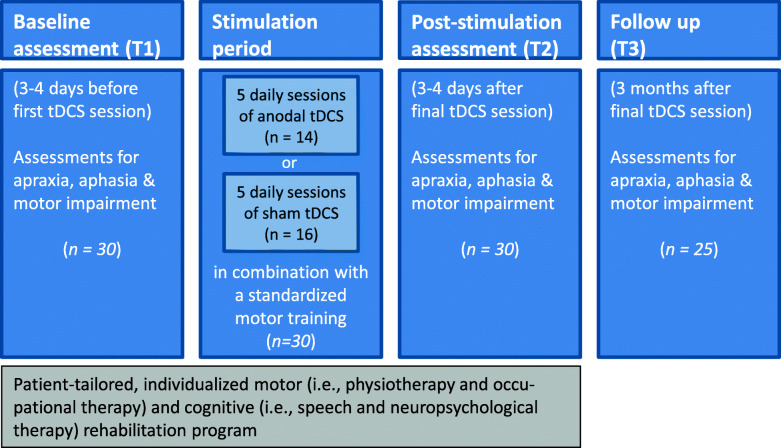
The study design consisted of a baseline assessment (3–4 days before first tDCS session), the stimulation period (comprising 5 daily session of either anodal or sham tDCS applied above left posterior parietal cortex, PPC, combined with motor training by three motor tasks, see text), and the post-stimulation assessment (3–4 days after final tDCS session). While all 30 LH stroke patients underwent these three study parts, 25 LH stroke patients performed a follow-up assessment, about 3 months after the final tDCS session. The apraxia and aphasia assessments focused on the Cologne Apraxia Screening (KAS) and the short version of the Aphasia Check-List (ACL-K). Grip force measures reflected the motor impairment. As depicted by the grey box, the baseline assessment, the stimulation period, and the post-stimulation assessment took place while the 30 patients were hospitalized for stroke rehabilitation undergoing a patient-tailored, individualized motor (i.e., physiotherapy and occupational therapy) and cognitive (i.e., speech and neuropsychological therapy) rehabilitation program

The study comprised a baseline assessment, the stimulation period, and the post-stimulation assessment. Furthermore, 25 (of the 30) LH stroke patients underwent a follow-up assessment (Fig. 2).

To extend previous findings of immediate effects of a single tDCS session on apraxic deficits in neurological patients [3], the primary outcome parameter was the change (post vs. pre-intervention) of the Cologne Apraxia Screening (KAS) total score after repetitive anodal (versus sham) tDCS applied over left PPC, at least 3 days after the last tDCS session. Taking into account the pivotal role of the left PPC in gesture imitation, the secondary outcome parameter was the change of the KAS imitation score at the post-stimulation assessment (compared to the baseline assessment).

The baseline assessment (taking place three or 4 days before the first tDCS session) focused on apraxic deficits, aphasia, and basic motor functions.

Apraxic deficits of the ipsilesional left hand were assessed with the Cologne Apraxia Screening (KAS, [44]). The KAS comprises two types of tasks (pantomime, imitation), each performed with two effectors (bucco-facial gestures, limb gestures). This factorial structure results in the following four KAS subtests (consisting of five items each): 1. pantomime of object use involving bucco-facial movements; 2. pantomime of object use involving limb movements; 3. imitation of bucco-facial gestures; 4. imitation of limb gestures. The test material consists of photos that either depict the object/tool or a woman showing the gesture, minimizing the influence of concurrent aphasic deficits [28, 45]. In the pantomime subtests, one or two points (depending on the complexity) are awarded for predefined features of the pantomime. For example, for the item “pantomiming the use of a toothbrush” the following movement features are scored with one point each: (i) the hand is almost closed to a fist, (ii) the hand is held laterally in front of the mouth, (iii) the mouth is slightly opened, and the teeth are shown, and (iv) circling/ pushing movements of the hand. When a given movement feature is absent, no point is given. For the evaluation of the imitation subtests, four points are given for the correct imitation on the first trial. If the first imitation trial fails, the stimulus photo is shown for a second time. Two points are given for a successful second trial. If the patient also fails on the second trial, no point is given. For each KAS item, the patient can maximally achieve four points. Therefore, a maximum score of 20 points can be achieved in each of the four subtests, and the KAS total score can be maximally 80 points. Based on the normative data of the KAS [13], a psychometric analysis revealed that a patient with a score of 76 or less is considered apraxic.

Furthermore, we examined the actual object use of the ipsilesional left hand with five single objects (hammer, toothbrush, pair of scissors, toy gun, and pencil eraser) and two multiple-object tasks (key & padlock, matchbox & candle [11];). However, in the current sample of stroke patients, object use deficits were rare (*n* = 2, see also [28, 29]). Note, that the two patients with impaired object use also performed below cut-off in the KAS and were thus classified as apraxic.

We assessed patients for aphasia using the short version of the Aphasia Check-List (ACL-K, [26]). Testing of the motor function of the affected, contralesional right hand comprised an assessment of the degree of paresis (paresis scale of the Medical Research Council (MRC), [34]) and the strength of grip force (using a vigorimeter of the company KLS martin). To account for pre-existing individual differences in muscle strength, we normalized the grip force of the *contra*lesional right hand by the grip force of the *ipsi*lesional left hand.

The stimulation period comprised five tDCS sessions on five consecutive days (Monday to Friday of the week following the baseline assessment; in two patients only four sessions could be performed (Tuesday to Friday) due to a public holiday). We placed the active electrode (35cm^2^) over the left PPC (i.e., position P3 of the 10/20 EEG-system using an EEG-cap to facilitate positioning). We fixed the return electrode (45cm^2^) in the right (contralesional) supraorbital position [3]. During (active or sham) stimulation, patients were told not to talk or read and they were free to keep their eyes open or closed. tDCS was applied following current safety guidelines [33], using a constant current of 2 mA for 10 min for anodal tDCS, which resulted in a calculated current density of approximately 0.057 mA/cm^2^. According to the study mode of the DC-Stimulator (neuroConn GmbH, Ilmenau, Germany), a short stimulation of 2 mA was applied for 20s between the fade-in and fade-out periods, which were identical to the anodal stimulation and lasted 6 s each. This procedure prevented any effective modulation of cortical excitability by tDCS [17] and ensured blinding of the patients, since any putative sensations associated with anodal tDCS (e.g., itching, tingling) would also occur for sham tDCS [19]. The investigator, who applied tDCS (JMA), was blinded for the type of tDCS (anodal or sham) with the help of the NeuroConn study mode, in which an individual code triggered either anodal or sham stimulation of a given patient by the pre-programmed tDCS study device.

To promote motor plasticity, anodal (or sham) tDCS was combined with a standardized motor training, which the patients performed on each day of the stimulation period before and after tDCS. This standardized motor training compromised the following three complex motor tasks: (i) the Jebsen Taylor Hand Function Test (JTHFT, [25]): The JHFT simulates activities of daily living (ADLs) performed with the ipsilesional left hand. It comprises a time-dependent functional assessment of seven subtests (copying a sentence, turning cards over, picking up small objects and placing them in a can, simulated feeding, stacking checkers, and moving large (light and heavy) objects from one location to another); (ii) the imitation test by De Renzi and colleagues [10]: The De Renzi test comprises 24 (12 meaningful, 12 meaningless) intransitive gestures, which have to be imitated (up to three times) with the ipsilesional left hand, and (iii) the Action Research Arm Test (ARAT, [46]): The ARAT comprises a wide range of upper-extremity functions of both hands/arms including grasping, lifting and moving objects of various sizes and performing gross upper-extremity movements.

The post-stimulation assessment was scheduled 3 to 4 days after the last tDCS session. As in the baseline assessment, apraxic deficits (KAS, De Renzi object use test), aphasia (ACL-K), and basic motor functions (MRC paresis scale, grip force) were examined. Baseline and the post-stimulation assessments were performed by JMA, who was blind to the type of tDCS-stimulation at the time of testing.

The baseline assessment, the stimulation period, and the post-stimulation assessment took place while the patients were hospitalized for stroke rehabilitation undergoing a regular patient-tailored rehabilitation program including physiotherapy, occupational therapy, speech therapy, and neuropsychological therapy.

Participants who agreed to a follow-up assessment (including the tests applied for the baseline and post-stimulation assessments, i.e., KAS, ACL-K, and grip force) were revisited at their homes about 3 months after the last tDCS session (mean: 100 ± 9 days). Follow-up assessments were performed by JMA (*n* = 4) or a research assistant (*n* = 21), who were blind to the type of tDCS-stimulation at the time of the follow-up assessment.

All assessments were videotaped to allow an independent performance evaluation by a second rater, which were contrasted with the initial ratings by JMA and objectified by means of the interrater reliability of the KAS measurements. Pearson correlation analysis revealed a high interrater agreement for the KAS total scores at baseline (*r* = 0.945, *p* < .001), post-stimulation (*r* = 0.984, *p* < .001), and follow-up (*r* = 0.844, *p* < .001).

### Statistical analysis

All statistical analyses were performed using IBM SPSS statistics version 24. Demographic and neuropsychological measures of the two stimulation groups were compared by two-sample t-tests, while their gender distribution was compared with Fisher’s exact test.

Separate univariate ANOVAs analyzed the amount of therapy during the stimulation period with the between-group factors APRAXIA (apraxic, non-apraxic) and STIMULATION (anodal tDCS, sham tDCS).

The *medium-term* effects of anodal versus sham tDCS applied above the left PPC on apraxia, aphasia, and paresis were operationalized as changes of the KAS total, pantomime and imitation scores (for the ipsilesional hand), the ACL-K scores, and the grip force measures (for the contralesional hand) between the baseline and post-stimulation assessments. These measures were analyzed by separate three-way repeated measure ANOVAs with the within-subject factor TIME (baseline vs. post-stimulation assessments) and the between-subject factors APRAXIA (apraxic, non-apraxic) and STIMULATION (anodal tDCS, sham tDCS).

The *long-term* stimulation effects on apraxia, aphasia, and paresis were operationalized as group differences of the KAS total, pantomime and imitation scores (for the ipsilesional hand), the ACL-K scores, and the grip force measures (for the contralesional hand) that were present at the follow-up. Also, these measures were analyzed by separate univariate ANOVAs with the between-group factors APRAXIA (apraxic, non-apraxic) and STIMULATION (anodal tDCS, sham tDCS).

## Results

Unless stated otherwise, we present all results as mean ± SD.

### Behavioral/neuropsychological assessment

The sham (*n* = 16) and anodal (*n* = 14) tDCS groups did not differ significantly concerning age, time post-stroke, and gender distribution as well as the severity of the initial apraxic, aphasic, and motor deficits at baseline (see Table 1 for all contrasted demographic and neuropsychological variables, all *p* > .1).

During their stay in the rehabilitation hospital (and thus also during the study period), patients received a regular, patient-tailored motor (i.e., physiotherapy and occupational therapy) and cognitive (i.e., speech and neuropsychological therapy) rehabilitation program. Accordingly, the amount of rehabilitation treatment varied among patients. On average, patients received 168.8 (± 63.7) minutes of motor therapy per day and 64.4 (± 46.7) minutes of cognitive therapy per day. For the amount of motor as well as cognitive therapy during the stimulation period, the univariate ANOVAs with the between-group factors APRAXIA (apraxic, non-apraxic) and STIMULATION (anodal tDCS, sham tDCS) revealed no significant effects (all *p* > .1), including the interaction APRAXIA x STIMULATION. Thus, the observed differential tDCS-effects (see below) are most likely not caused by differences in the rehabilitation program during the study period.

### Medium-term effects of left parietal anodal tDCS on apraxia

Thirty patients with LH stroke entered the 3-way-ANOVAs evaluating the effect of anodal (versus sham) tDCS applied over left PPC (combined with motor training) on the baseline and post-stimulation apraxia scores.

**Fig. 3:**
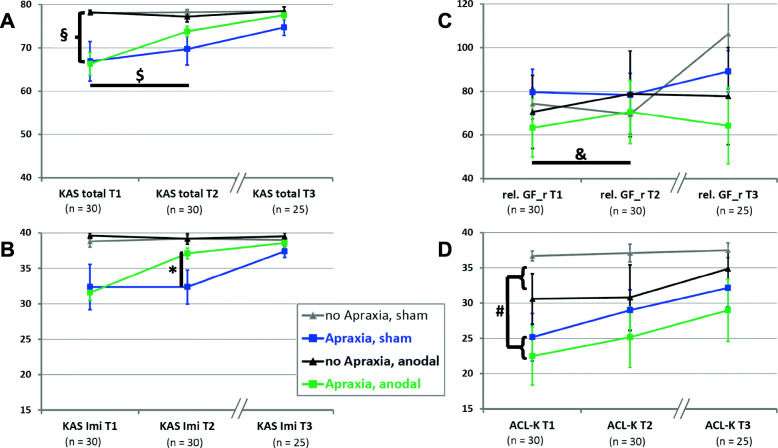
Graphical illustration of the Cologne Apraxia Screening (KAS) total score (**a**, upper, left panel), the KAS imitation subscore (**b**, lower, left panel), the relative grip force of the contra-lesional, right hand (**c**, upper, right panel), and the scores of the short version of the Aphasia Check-List (ACL-K, **d**, lower, right panel) in the four patient groups with LH stroke across the three assessments (baseline assessment [T1, *n* = 30], post-stimulation assessment [T2, *n* = 30], and follow-up assessment [T3, *n* = 25]). LH stroke patients with apraxia (squares) undergoing anodal tDCS (green, A+ anodal) or sham tDCS (blue, A+ sham), LH stroke patients without apraxia (triangles) undergoing anodal tDCS (black, A- anodal) or sham tDCS (grey, A- sham). Displayed are the means and the standard error of the mean (SEM). **a**. Apraxic patients scored significantly lower on the KAS than patients without apraxia (§, significant main effects of APRAXIA F (1,26) = 6.6, *p* < .05). Furthermore, there was a significant interaction APRAXIA by TIME ($, F (1,26) = 6.7, *p* < .05), indicating that the KAS total scores of apraxic patients (KAS total score at baseline: 66.7 ± 12.1, KAS total score at post-stimulation: 71.6 ± 9.3) improved significantly more from baseline to post-stimulation than those of the non-apraxic patients (KAS total score at baseline: 78.1 ± 8.8, KAS total score at post-stimulation: 77.7 ± 2.2; t (28) = − 2.4, *p* < .05). **b**. For the scores of the imitation subtests of the KAS (KAS imi), the asterix (*) indicates the significant 3-way-interaction APRAXIA by TIME by STIMULATION (F (1,26) = 4.6, *p* < .05): The KAS imitation scores (maximum of 40 points) of apraxic patients who underwent anodal tDCS (green squares) improved significantly more from baseline to post-stimulation (from 31.6 ± 3.3 to 37.1 ± 2.3 points) than those of the apraxic patients undergoing sham tDCS (blue squares, from 32.4 ± 10.6 to 32.4 ± 7.9 points; t (18) = − 2.8, *p* < .05), while there was no significant modulation by stimulation nor relevant changes with time for the non-apraxic patients (non-apraxic, anodal [black triangles]: 39.6 ± 0.9 to 39.2 ± 1.8 points; non-apraxic, sham [grey triangles]: 38.8 ± 1.8 to 39.2 ± 1.1 points; t (8) = 0.65, *p* = 0.535). **c**. Independent of apraxia, there was a significant interaction TIME by STIMULATION (&, F (1,26) = 4.9, *p* < .05), indicating that the grip force levels of the contralesional, right hand significantly improved from baseline to post-stimulation in the stroke patients who underwent anodal tDCS, while no relevant changes were observed for the sham group (anodal tDCS group [green and black lines]: from 65.8 ± 37.9% to 73.5 ± 42.1%; sham tDCS group [blue and grey lines]: from 77.9 ± 29.7% to 75.6 ± 29.1%). Rel. GF_r = relative grip force of the contra-lesional right hand (in relation to the ipsi-lesional left hand, in %). **d**. There were no significant differential effects of tDCS on the ACL-K-scores, but a main effect of APRAXIA (#, F (1,26) = 4.6, *p* < .05), which indicated more severe aphasic deficits in the apraxic patients (squares, 25.5 ± 11.5) compared to patients without apraxia (triangles, 33.8 ± 6.3)

For the KAS total score (maximum of 80 points), the ANOVA with the factors APRAXIA (apraxic vs. non-apraxic, i.e., KAS scores below vs. above cut-off), STIMULATION (anodal vs. sham tDCS), and TIME (baseline vs. post-stimulation) revealed significant main effects of APRAXIA (i.e., apraxic patients scored significantly lower on the KAS than patients without apraxia; F (1,26) = 6.6, *p* < .05) and TIME (i.e., from baseline to post-stimulation, stroke patients overall improved in their KAS total scores; F (1,26) = 4.9, *p* < .05; Fig. 3a). Furthermore, there was a significant interaction APRAXIA by TIME (F (1,26) = 6.7, *p* < .05), indicating that the KAS total scores of apraxic patients (KAS total score at baseline: 66.7 ± 12.1, KAS total score at post-stimulation: 71.6 ± 9.3; t (19) = − 3.2, *p* < .01) improved significantly more from baseline to post-stimulation than those of the non-apraxic patients (KAS total score at baseline: 78.1 ± 0.8, KAS total score at post-stimulation: 77.7 ± 2.2; t (9) = 0.8, *p* > 0.4; Fig. 3a). However, the 3-way-interaction of APRAXIA by TIME by STIMULATION failed to reach significance (F (1,26) = 1,9, *p* = .18). Thus, the primary outcome of the study (i.e., anodal tDCS induced improvement of the KAS total score) failed to reach significance.

Importantly, we found a significant 3-way-interaction APRAXIA by TIME by STIMULATION for the scores of the imitation subtests of the KAS (F (1,26) = 4.6, p < .05; Fig. 3b), indicating that the KAS imitation scores (maximum of 40 points) of apraxic patients who underwent anodal tDCS above the left PPC improved significantly more from baseline to post-stimulation (from 31.6 ± 3.3 to 37.1 ± 2.3 points, t (8) = − 3.7, *p* < .01) than those of the apraxic patients undergoing sham tDCS (from 32.4 ± 10.6 to 32.4 ± 7.9 points, t (10) = 0, *p* = 1), while there was no significant modulation by stimulation nor relevant changes with time for the non-apraxic patients (non-apraxic, anodal: 39.6 ± 0.9 to 39.2 ± 1.8 points, t (4) = 0.41, *p* > .7; non-apraxic, sham: 38.8 ± 1.8 to 39.2 ± 1.1 points, t (4) = − 0.54, *p* > .6; see Fig. 3b). In contrast, for the scores of the pantomime subtest of the KAS, no significant effects were found (*p* > .05).

Further examination of the differential stimulation-related improvements in imitation performance revealed that apraxic patients, who underwent anodal tDCS, achieved similar KAS imitation scores as the non-apraxic patients already at the post-stimulation assessment (*p* = .102), while the KAS imitation scores between these groups clearly differed at the time of baseline assessment (t (12) = 5.3, *p* < .001).

### Medium-term effects of left parietal anodal tDCS on aphasia and grip force

To evaluate putative tDCS effects on the aphasic deficits of apraxic and non-apraxic patients and to assess whether improvements in apraxia scores were paralleled by changes in aphasia severity [39], we computed an ANOVA with the factors APRAXIA (apraxic vs. non-apraxic), STIMULATION (anodal vs. sham tDCS), and TIME (baseline vs. post-stimulation assessment) for the ACL-K-scores (Fig. 3d). Except for a main effect of APRAXIA (F (1,26) = 4.6, *p* < .05), which indicated more severe aphasic deficits in the apraxic patients (25.5 ± 11.5) compared to patients without apraxia (33.8 ± 6.3; cf. [45]), no significant effects were observed (all *p* > .06, Fig. 3d). Furthermore, we computed a complementary ANOVA for the ACL-K-scores with the factors APHASIA (aphasic vs. non-aphasic), STIMULATION (anodal vs. sham tDCS), and TIME (baseline vs. post-stimulation assessment). This analysis revealed the two significant main effects of TIME (F (1,26) = 9.2, *p* < .01), indicating an overall improvement of the ACL-K-scores between the two assessments (baseline: 27.2 ± 11.0, post-stimulation: 29.5 ± 10.5), and APHASIA (F (1,26) = 45.7, *p* < .001), indicating (i.e., by definition) lower ACL-K-Scores in the aphasic (18.3 ± 8.8) than non-aphasic (36.1 ± 1.8) patients. The other main effects and interactions did not reach significance (all *p* > .08).

To evaluate whether the improvements of apraxia scores (as a marker for stroke-related motor-*cognitive* deficits assessed with the ipsi-lesional, left hand) are paralleled by changes in *basic* motor functions after stroke (here: grip force levels of the contra-lateral, right hand), we computed for the grip force measures of the contralesional, right hand (normalized by the grip force of the ipsilesional, left hand to account for pre-existing individual differences in muscle strength) an ANOVA with the factors APRAXIA (apraxic vs. non-apraxic), STIMULATION (anodal vs. sham tDCS), and TIME (baseline vs. post-stimulation assessment). This ANOVA revealed that - irrespective of the presence/absence of apraxia - grip force levels of the *contra*lesional right hand significantly improved from baseline to post-stimulation in the stroke patients who underwent anodal tDCS, while no changes were observed for the sham group (anodal tDCS group: from 65.8 ± 37.9% to 73.5 ± 42.1%, t (13) = − 2.52, *p* < .05; sham tDCS group: from 77.9 ± 29.7% to 75.6 ± 29.1%, t (15) = 0.95, *p* > .3; significant interaction TIME by STIMULATION: F (1,26) = 4.9, *p* < .05, Fig. 3c). This finding indicates a facilitatory effect of anodal tDCS on an essential primary motor function independent of apraxia. All other comparisons did not reveal any significant changes in grip force (p > .3). Note that all apraxia assessments (e.g., KAS) were performed with the ipsilesional, left hand.

An additional ANOVA with the between-subject factors PARESIS (patients with relevant paresis (i.e., MRC grade ≤ 3) vs. patients without relevant paresis (i.e., MRC grade ≥ 4)) and STIMULATION (anodal vs. sham tDCS) and the within-subject factor TIME (baseline vs. post-stimulation assessment) revealed that - by definition - grip force levels of the *contra*lesional, right hand were lower in stroke patients with (11.7 ± 19.9%) versus without (84.4 ± 19.8%) paresis (main effect of PARESIS: F (1,26) = 43.9, *p* < .001). Again, the other main effects and interactions did not reach significance (all *p* > .2).

### Long-term effects of left parietal anodal tDCS on apraxia, aphasia, and paresis

Twenty-five (of the initial 30) patients with LH stroke were available for the follow-up assessment after 3 months (100 ± 9 days). We entered the scores derived from the comprehensive follow-up assessment into ANOVAs with the between-group factors APRAXIA (apraxic vs. non-apraxic) and STIMULATION (anodal vs. sham tDCS). For all applied tests (including the KAS, ACL-K, and grip force measures), no significant main effects or interactions were observed (all *p* > .1, see also Fig. 3), indicating that the current LH stroke patients showed comparable test performance at the follow-up assessment independent of the initial presence of apraxic deficits or the applied stimulation protocol (anodal vs. sham tDCS). Thus, there were no significant effects of anodal tDCS on apraxia after 3 months.

Note that the KAS imitation scores at post-stimulation assessment (37.11 ± 2.26) of the apraxic patients, who had received anodal tDCS, were comparable to the KAS imitation scores at the follow-up assessment (37.4 ± 2.67) of the apraxic patients who had received sham tDCS (*p* = .804, see Fig. 3). In other words, apraxic patients, who underwent anodal tDCS, achieved KAS imitation scores already after about 10 days that were comparable to those achieved by the apraxic patients undergoing sham tDCS after about 100 days.

## Discussion

In this double-blinded, sham-controlled tDCS pilot study we observed that in LH stroke patients the recovery from apraxic imitation deficits is significantly expedited by repetitive anodal tDCS (relative to sham tDCS) applied over left posterior parietal cortex (PPC) combined with standardized motor training. In fact, after five consecutive tDCS-sessions, the imitation performance of the initially apraxic LH stroke patients undergoing anodal parietal stimulation recovered so much that it was comparable to that of the non-apraxic patients. However, the primary outcome of the study (i.e., tDCS induced improvement of the KAS total score) failed significance, and there was no significant effect of anodal tDCS on apraxia after 3 months.

The current study confirms but, importantly, extends previous findings of positive *single* session tDCS effects on apraxic deficits in neurological patients [3] in various ways. Unique features of the current study are the use of a control patient group, double-blinding of tDCS conditions, and follow-up assessments. In contrast to previous tDCS studies (e.g., *n* = 6 stroke patients in Bolognini et al. [3]), the current pilot study employed 30 patients. Nevertheless, future studies need to include larger sample sizes, since the effect sizes of tDCS interventions in stroke patients are weak to moderate [6, 27]. Importantly, while previous studies revealed only immediate effects directly after tDCS (i.e., minutes after the end of stimulation), the current data show a significant effect on apraxic imitation deficits lasting at least 3 days after the last session of parietal anodal tDCS. Moreover, the current tDCS effects were controlled for unspecific effects by employing a control patient group.

Furthermore, the enhanced recovery of grip force of the contralesional right hand in the LH stroke patients undergoing anodal but not sham tDCS (independent of apraxia) suggests an additional positive modulation of an essential motor function by anodal tDCS applied over left PPC and thereby extends previous findings of studies modulating the primary motor cortex of the affected hemisphere [2, 24, 42]. This novel finding warrants further investigation in future studies.

In contrast to imitation, there was no significant effect of tDCS on pantomime performance. This lack of an effect can be explained by the notion that over and above an involvement of the left parietal cortex many different areas within a distributed left-hemispheric network are critically involved in pantomiming the use of objects [32]. Thus, the current stimulation above the left PPC was able to ameliorate imitation performance due to the essential role of the left PPC in the processing of gestures for imitation [1, 43], but failed to improve pantomiming of object use, since further relevant areas for pantomiming (e.g., ventral stream areas [23] or frontal regions [45]) were outside the stimulated brain region (i.e., left PPC).

Despite relevant aphasic deficits (see Table 1), anodal parietal tDCS improving apraxic deficits did not have concurrent effects on aphasia in the current patient sample. The most parsimonious explanation for this observation is that those brain regions (e.g., frontal cortex [30], motor cortex [31], temporal cortex [18]) that have been targeted by tDCS studies on aphasic deficits after stroke [15] were outside the current stimulation focus. Notably, Bolognini and colleagues also did not find an effect of tDCS delivered to the left PPC on phonemic fluency in their six stroke patients [3].

Concerning the specificity of the tDCS effects on apraxic deficits, note that (anodal and sham) tDCS was combined with a standardized motor training consisting of three complex motor tasks that the patients performed on each day of the stimulation period before and after tDCS. Thus, our data suggest that the combination of anodal tDCS applied over left PPC and a standardized motor training expedited the recovery from apraxic imitation deficits. This finding supports the current notion that the specificity of tDCS in a given domain may result from the task-specific activation of the critical nodes within a domain-specific network (here: praxis network) already activated by anodal tDCS [9, 16].

## Limitations

The fact that study participants were not randomly, but rather consecutively assigned to the anodal and sham tDCS groups by a person who was not involved in the assessments or tDCS stimulation of the LH stroke patients is a limitation of the current pilot study. Therefore, aspects that were not captured by the initial clinical or neuropsychological assessments (e.g., level of motivation) could have influenced the results. However, at baseline the two patient groups were matched for essential parameters: age, time post-stroke, severity of apraxia and aphasia as well as basic motor (dys-)function (see Table 1). Nevertheless, forthcoming tDCS studies on stroke recovery should employ randomization procedures. Note that the current study is the first that employed a control group with apraxic LH stroke patients (cf. [3]).

In healthy young participants, recent studies reported compromised blinding of tDCS conditions [21, 40]. Thus, the lack of a systematic assessment of blinding efficacy constitutes a limitation of our study. However, the participants of the current pilot study were LH stroke patients suffering from cognitive (e.g., apraxic, aphasic) deficits of different severity, which most likely have reduced their ability to distinguish real from sham tDCS, as has previously shown in clinical populations [19]. Nevertheless, future clinical tDCS studies should include a systematic assessment of blinding efficacy (e.g., by using questionnaires as in [4]) and employ optimized sham tDCS protocols [17].

## Conclusions

Our data show for the first time that repetitive, anodal tDCS over left PPC combined with a standardized motor training expedites recovery from imitation deficits in LH stroke patients with apraxia. However, the primary outcome of the study (i.e., anodal tDCS induced improvement of the total apraxia score) failed significance, and there was no significant tDCS effect on apraxia after 3 months. Nevertheless, based on these findings, a randomized controlled trial with an even larger sample of LH stroke patients suffering from (imitation) apraxia is warranted.

## Data Availability

The datasets used and/or analysed during the current study are available from the corresponding author on reasonable request.

## Abbreviations

ACL-K: Aphasia Check-List (short version)
HADS: Hospital Anxiety and Depression Scale
KAS: Cologne Apraxia Screening
LH: Left hemisphere
LQ: Laterality Quotient as assessed by the Edinburgh handedness inventory
MMSE: Mini-Mental State Examination
MRC: Medical Research Council
mRS: Modified Rankin Scale
PPC: Posterior parietal cortex
tDCS: Transcranial direct current stimulation
